# Reliable Classifier to Differentiate Primary and Secondary Acute Dengue Infection Based on IgG ELISA

**DOI:** 10.1371/journal.pone.0004945

**Published:** 2009-04-02

**Authors:** Marli Tenório Cordeiro, Ulisses Braga-Neto, Rita Maria Ribeiro Nogueira, Ernesto T. A. Marques

**Affiliations:** 1 Virology and Experimental Therapy Laboratory, Aggeu Magalhães Research Center, Fiocruz, Recife, Pernambuco, Brazil; 2 Central Laboratory of Public Health, Secretaria de Saúde do Estado de Pernambuco, Recife, Brazil; 3 Laboratory of Flavivirus, Institute Oswaldo Cruz, Fiocruz, Rio de Janeiro, Brazil; 4 Department of Electrical and Computer Engineering, Texas A&M University, College Station, Texas, United States of America; 5 Department of Medicine, Division of Infectious Diseases, The Johns Hopkins School of Medicine, Baltimore, Maryland, United States of America; 6 Department of Pharmacology and Molecular Sciences, The Johns Hopkins School of Medicine, Baltimore, Maryland, United States of America; Singapore Immunology Network, Singapore

## Abstract

**Background:**

Dengue virus infection causes a wide spectrum of illness, ranging from sub-clinical to severe disease. Severe dengue is associated with sequential viral infections. A strict definition of primary versus secondary dengue infections requires a combination of several tests performed at different stages of the disease, which is not practical.

**Methods and Findings:**

We developed a simple method to classify dengue infections as primary or secondary based on the levels of dengue-specific IgG. A group of 109 dengue infection patients were classified as having primary or secondary dengue infection on the basis of a strict combination of results from assays of antigen-specific IgM and IgG, isolation of virus and detection of the viral genome by PCR tests performed on multiple samples, collected from each patient over a period of 30 days. The dengue-specific IgG levels of all samples from 59 of the patients were analyzed by linear discriminant analysis (LDA), and one- and two-dimensional classifiers were designed. The one-dimensional classifier was estimated by bolstered resubstitution error estimation to have 75.1% sensitivity and 92.5% specificity. The two-dimensional classifier was designed by taking also into consideration the number of days after the onset of symptoms, with an estimated sensitivity and specificity of 91.64% and 92.46%. The performance of the two-dimensional classifier was validated using an independent test set of standard samples from the remaining 50 patients. The classifications of the independent set of samples determined by the two-dimensional classifiers were further validated by comparing with two other dengue classification methods: hemagglutination inhibition (HI) assay and an in-house anti-dengue IgG-capture ELISA method. The decisions made with the two-dimensional classifier were in 100% accordance with the HI assay and 96% with the in-house ELISA.

**Conclusions:**

Once acute dengue infection has been determined, a 2-D classifier based on common dengue virus IgG kits can reliably distinguish primary and secondary dengue infections. Software for calculation and validation of the 2-D classifier is made available for download.

## Introduction

Dengue virus (DENV) is a member of the family *Flaviviridae*, genus *Flavivirus*, with four antigenically distinct serotypes (DENV-1 to DENV-4). Infection with this virus is a growing public health concern in tropical and subtropical regions of the world, with an estimated incidence of 50–100 million cases per year [1]. Dengue virus infection in humans causes a large spectrum of illness ranging from mild sub-clinical disease to a severe and occasionally fatal hemorrhagic clinical form, the dengue hemorrhagic fever (DHF) [2]. Severe complications of dengue infections such as DHF are mainly associated with sequential infection [3], [4]. The lack of adequate tools to predict whether a patient infected with dengue virus will progress with the benign form or with life-threatening disease has often resulted in a large number of unnecessary and costly hospitalizations, which during dengue outbreaks have led to a public health crisis by creating a shortage of hospital beds [5]. Consequently, the differentiation of primary from secondary infection may be of great prognostic value for dengue patients, particularly children and the elderly, in whom a secondary dengue infection is more likely to result in DHF [4], [5]. Also, for epidemiological purposes, it is important to characterize the dengue serological immune response during dengue outbreaks [5].

There is no doubt that clinical observation is the most important criterion for dengue diagnosis; nevertheless, definitive diagnosis of the disease requires laboratory confirmation [6], [7], [8]. ELISA-based detection of specific antibodies (both IgM and IgG) to the four dengue serotypes is valuable for the diagnosis of acute infection and for detection of previous exposure to dengue virus [8], [9]. The hemagglutination inhibition (HI) test [10], based on antibody titering of paired serum specimens and recommended by the World Health Organization (WHO) [7], is the method that has most frequently been used for serologic classification of dengue infections. However, this assay is time-consuming and cross-reactions among *Flavivirus* have been noted [6]. The plaque reduction neutralizing test (PRNT) [11] may be also used, but this assay is difficult for most laboratories to perform, and the fact that the results take several days to obtain tends to limit its clinical usefulness.

Thus, an accurate, timely and affordable assay that could be used to characterize the serologic response to DENV infection is clearly desirable. As an alternative to the HI test, several laboratories have developed and evaluated ELISA-format tests to detect IgG antibodies [12], [13]. The IgG ELISA has the advantage of being easier to perform, as well as being suitable for surveillance and large-scale studies [14]. A number of commercial and standardized ELISA kits for both IgM and IgG antibody detection have also become available. The commercial IgG-capture ELISA kits that have been evaluated have shown good correlation with the HI assay [15]. However, the ability of both in-house ELISAs and commercial kits to classify accurately primary and secondary dengue infections still needs to be validated with standard reference samples.

This report shows how to design a linear 2-dimensional (2-D) classifier to assign primary and secondary dengue infection status to patients based on their IgG response, as measured by assays of samples taken on different days after onset of symptoms. This study is based on a set of IgG antibody data for patients from a well-characterized dengue cohort in the city of Recife, Brazil [16], where a commercial dengue IgG-ELISA kit is routinely used. In this kit the IgG-based classification of dengue infection is based on the use of a constant cut-off value to discriminate between the two types of infection, regardless of disease stage (as measured here by the number of days from the onset of symptoms). In the present study, we describe how to develop a reliable 2-D classifier that takes into account the disease stage and the IgG antibody level, and we demonstrate that this approach shows excellent performance with independent test data obtained from patients with independently verified primary or secondary dengue infections.

## Materials and Methods

### Study population and specimen collection

Volunteers were recruited among subjects with more than five years of age who were admitted to one of three hospitals in the city of Recife —Instituto Materno Infantil de Pernambuco (IMIP), Hospital Esperança (HE) and Hospital Santa Joana (HSJ) — under suspicion of an acute dengue infection. Disease day 1 was the day of onset of symptoms, as reported by the patient. Blood samples were collected at the time of the first visit to the hospital. The patients had from two to five blood samples taken, on various days after the onset of disease. All first serum samples were evaluated using the standard tests: virus isolation, RT-PCR and serology (IgM and IgG). Serology only was carried out on all subsequent samples. Highly-experienced technicians performed all the assays in a blinded fashion, before any assignment of primary or secondary infection was made. Dengue cases were laboratory-confirmed by virus isolation and/or viral RNA detection by RT-PCR and/or by a positive anti-dengue, IgM-capture ELISA. A subset of samples collected during the two first years of the cohort (2004–2005) was employed. The complete clinical-epidemiological description of the cohort is described elsewhere [16]. The demographic description of the subjects in this study is presented in Table 1.

**Table 1:** Demographic of the patients. Primary and Secondary infection Information is based on the CPqAM classification criteria.

SUBJECT AGE Years	GENDER			TYPE OF INFECTION	
	Male	Female	Total	Primary	Secondary
**5–9**		1	1		1
**10–14**	1	3	4	2	2
**15–24**	9	8	17	14	3
**25–34**	13	16	29	15	14
**35–44**	17	11	28	16	12
**≥45**	14	16	30	10	20
**TOTAL**	**54**	**55**	**109**	**57**	**52**

### Reference Standard (“Recife” method)

During the 2004–2005 period, the cohort had 230 subjects enlisted with confirmed dengue cases. From those 230 cases, 109 where unambiguously classified as primary or secondary infections based on a series of multiple standard methods (see below), and a total of 322 blood samples were obtained from these subjects. All the reference samples were strictly assigned by the authors according to the following criteria: 1) Primary infection (P) was characterized by absence of dengue specific IgG antibodies in the acute serum sample and presence of anti-dengue IgM, virus isolation and/or viral RNA detection, followed by the presence of anti-dengue IgG in convalescent serum samples; 2) Secondary infection (S) was characterized by presence of specific anti-dengue IgG in the acute sample and absence of anti-dengue IgM, associated with a positive RT-PCR and/or virus isolation; followed by the presence of anti-dengue IgM in convalescent serum samples. All unambiguously assigned samples were included in this study; no exclusions were made. The remaining 111 dengue cases could not be rigorously defined as primary or secondary dengue infections based on the criteria above and did not participate in the development of the classifier.

### Ethical considerations

Written consent to participate in the study was obtained from each patient (or the patient's guardian) after a full explanation of the study was provided. All data were handled confidentially and anonymously. This study was reviewed and approved by the ethics committee of the Brazilian Ministry of Health (N° 4909 CONEP) and The Johns Hopkins University School of Medicine internal review board (# 03-08-27-01).

### Cohort serum collection

Blood samples were collected into 10 ml Vacutainer® tubes (Becton Dickinson, Franklin Lakes, NJ). Serum was separated by centrifuging the tubes at 1,600×g for 10 min. Samples (1 ml per tube) were stored in two cryovials at −80°C and −20°C for later use in virus isolation, RT-PCR and serology.

### Virus isolation and identification

For DENV isolation, serum samples were inoculated onto a monolayer of C6/36 cells [17]. Cells were harvested after 10–14 days of incubation, and checked for the presence of virus by immunofluorescence assay. The dengue virus was identified with serotype-specific monoclonal antibodies as described by Gubler et al. [18].

### Reverse transcriptase- polymerase chain reaction (RT-PCR)

Viral RNA was extracted from serum samples using a QIAquick PCR Purification kit (QIAGEN Inc. Valencia, CA). A two-step nested RT-PCR was carried out on all initial serum samples according to Lanciotti et al. [19]. Negative and positive controls were included in all steps. A purified and quantified dengue virus control was added to the PCR test to confirm the limit of detection of each assay of 10 genomic copies.

### Serology

#### IgM ELISA

A total of 322 serum samples were used for IgM and IgG antibody detection. An anti-dengue, IgM-capture ELISA based on the viral envelop protein (Bio-Manguinhos, Fundação Oswaldo Cruz, Brazil) was performed according to the manufacturer's instructions. Results were interpreted as negative or positive according to the assay manual.

#### IgG ELISA

An anti-dengue, IgG-capture ELISA (PanBio, Pty., Ltd., Brisbane, Australia) was performed according to the recommended guidelines. In brief, 100 µl/well of patient or control sera, diluted 1∶100 in the reagent provided, was added to the assay plate, containing a combination of the envelope antigens (DENV-1, 2, 3 and 4) attached to its surface. After incubation, the residual serum was removed by washing, and 100 µl/well of peroxidase-conjugated anti-human IgG was added. After incubation and washing steps, 100 µl/well of the substrate system (tetramethylbenzidine / hydrogen peroxidase) was added. The reaction was stopped by the addition of 100 µ/well of 1 M phosphoric acid and the absorbance was read at 450 nm. The results were calculated and interpreted according to manufacturer's instructions. Anti-dengue IgG PanBio units were calculated by dividing the sample absorbance by the cut-off value and then multiplying this value by 10 (IgG Reference Unit). Results of PanBio Units were interpreted as follow: >11, positive; <9, negative; and 9–11, equivocal. The cross-reactivity of the IgG-ELISA PanBio was investigated in dengue-IgG negative samples from 32 yellow fever vaccinees. There was no detectable dengue seroconvertion due to 17DD vaccination among these individuals, indicating very low cross-reactivity of the PanBio kit with yellow fever vaccinees (Table S1). However, six of the 32 dengue-negative IgG seroconverted 45 to 90 days later as a result of natural dengue infection, as determined by the presence of dengue specific IgM.

#### In house IgG-ELISA (“Rio” method)

The in-house IgG-ELISA was conducted at the Flavivirus Laboratory of the Instituto Oswaldo Cruz (IOC), Fiocruz (Rio de Janeiro, Brazil); the protocol used for the characterization of dengue immune response was previously described by Miagostovich et al [13]. Briefly, plates (96-well (8×12) microtiter plate, Immulon II, Dynatech, Inc., McLean, VA) were covered with 100 µl/well of hyper immune ascitic fluid (a mixture of anti-DENV-1, 2, 3 and 4 in equal parts) diluted in 0.1 M sodium carbonate buffer, pH 9.6, and were incubated overnight at 4°C. After washing, wells were blocked by filling with standard diluents (PBS pH 7.4/0,05% Tween/3% normal goat serum) and incubated for 1 h at 37°C. Seventy five microliters of 32 hemagglutinating units of purified virus antigen mix (DENV-1, 2, 3 and 4), diluted in standard diluents, was applied to each well and plates were incubated for 1 h at 37°C. After being washed three times in PBS, 100 µl of serum diluted 1∶40 in PBS/Tween/3% non-fat dry milk (NFDM diluents) was added to the first well in each column and 75 µl of the same diluents was added to the remaining wells. Four-fold dilutions were carried out to the eighth well in each column by transferring and mixing 25 µl. Plates were incubated for 1 h at 37°C, washed NFDM diluents was added. After incubating for 1 h at room temperature, plates were washed six times and 100 µl of substrate (ABTS) were added to each well. Plates were incubated at room temperature for 30 minutes, for color development, and the absorbance was read at 450 nm. Each plate contained a negative serum control, and the absorbance of each dilution was subtracted from the corresponding dilution of each test sample. According to this IgG-ELISA criteria, the immune response is defined as primary when acute-phase serum samples obtained before day 5 of illness have IgG antibody titers <1∶160 and convalescent-phase sera have titers ≤1∶ 40,960. Infections are defined as secondary when IgG titers are ≥1∶160 in the acute-phase serum and ≥1∶163,840 in convalescent-phase samples. The correlation of titers and serologic interpretations between IgG-capture ELISA and the hemagglutination inhibition assay (HI) were applied to validate the in-house IgG-capture ELISA.

#### Hemagglutination Inhibition assay (“HI” method)

The HI test was performed aiming to classify the patient's immune response and compare the results to the other two methods above. The HI assay [10] modified to a microtiter plate format was performed on paired serum samples from all the 50 cases used as the independent test set. Antigens of DENV-1, -2, -3 and Yellow Fever, provided by the Evandro Chagas Institute (Belém-Pará), Brazil, were used. The dengue immune response was classified according to WHO criteria [7]: cases with no HI antibodies (<1∶20) in acute phase serum collected before the fourth day of disease and convalescent phase serum samples with an HI titer <1∶1280 were classified as primary infection. Infections were classified as secondary in patients with HI antibody titers of 1∶20 or greater in the acute phase serum and a convalescent HI antibody titer greater than or equal to 1∶2560 [7]. A summary of the results of all the tests performed on the independent set of standard samples is shown in Table 2.

**Table 2:** Characterization of the “Standard Test Set” samples used for test validation.

Patient and sample N°	N° days	Diagnostic Tests			HI test				In house IgG ELISA (“Rio” method)	Classification/ Type of Infection
		IgM	RT- PCR	IgG RU	DENV1	DENV2	DENV3	YFV		
**262 S1**	2	POS	NEG	31	1∶160	1∶160	1∶320	1∶160	1∶2560	S
**262 S4**	15	POS		32	≥1∶2560	≥1∶2560	≥1∶2560	≥1∶2560	1∶40960	
**301 S1**	4	POS	NEG	25	≥1∶2560	1∶640	1∶80	1∶1280	1∶2560	S
**301 S3**	17	POS		29	≥1∶2560	≥1∶2560	1∶640	≥1∶2560	1∶10240	
**329 S1**	2	POS	NEG	38	≥1∶2560	≥1∶2560	1∶640	≥1∶2560	1∶10240	S
**329 S4**	11	POS		40	≥1∶2560	≥1∶2560	≥1∶2560	≥1∶2560	1∶163840	
**331 S1**	4	POS	D3	22	1∶1280	1∶640	1∶80	1∶1280	1∶2560	S
**331 S4**	14	POS		44	≥1∶2560	≥1∶2560	1∶1280	≥1∶2560	1∶163840	
**332 S1**	4	POS	NEG	38	≥1∶2560	1∶640	1∶80	1∶640	1∶10240	S
**332 S4**	11	POS		38	≥1∶2560	≥1∶2560	≥1∶2560	≥1∶2560	1∶163840	
**339 S1**	6	POS	D3	2	<1∶20	<1∶20	1∶40	<1∶20	1∶40	P
**339 S3**	14	POS		27	1∶40	1∶40	1∶320	1∶20	1∶10240	
**348 S1**	5	NEG	NEG	29	1∶1280	1∶640	1∶160	1∶640	1∶10240	S
**348 S3**	23	POS		37	≥1∶2560	≥1∶2560	≥1∶2560	≥1∶2560	1∶40960	
**355 S1**	4	NEG	D3	2	<1∶20	<1∶20	<1∶20	<1∶20	<1∶40	P
**355 S4**	15	POS		19	1∶80	1∶40	1∶320	<1∶20	1∶2560	
**358 S1**	5	POS	D3	3	<1∶20	<1∶20	1∶40	<1∶20	1∶160	P
**358 S3**	11	POS		25	1∶20	1∶40	1∶160	1∶20	1∶2560	
**361 S1**	4	NEG	D3	33	1∶1280	1∶640	1∶40	1∶80	1∶10240	S
**361 S4**	17	POS		40	≥1∶2560	≥1∶2560	≥1∶2560	≥1∶2560	1∶163840	
**370 S1**	7	POS	NEG	1	<1∶20	<1∶20	1∶80	1∶20	<1∶40	P
**370 S3**	12	POS		24	1∶80	1∶80	1∶640	1∶80	1∶640	
**372 S1**	3	NEG	D3	1	<1∶20	<1∶20	<1∶20	<1∶20	<1∶40	P
**372 S5**	30	POS		30	1∶80	1∶40	1∶640	1∶40	1∶160	
**382 S1**	5	POS	D3	27	≥1∶2560	1∶640	1∶160	1∶640	1∶10240	S
**382 S4**	12	POS		41	≥1∶2560	≥1∶2560	≥1∶2560	≥1∶2560	1∶163840	
**400 S1**	5	NEG	D3	1	<1∶20	<1∶20	<1∶20	<1∶20	<1∶40*	P
**400 S3**	15	POS		33	1∶80	1∶80	1∶640	1∶80	<1∶40	
**403 S1**	7	NEG	D3	46	1∶40	1∶20	<1∶20	1∶40	1∶10240	S
**403 S3**	13	NEG		39	≥1∶2560	≥1∶2560	1∶1280	1∶1280	1∶163840	
**406 S1**	6	NEG	D3	2	<1∶20	<1∶20	<1∶20	<1∶20	<1∶40	P
**406 S3**	28	POS		20	1∶80	1∶80	1∶640	1∶80	1∶10240	
**418 S1**	4	NEG	D3	14	1∶40	1∶20	<1∶20	1∶40	1∶160	S
**418 S4**	11	POS		18	≥1∶2560	≥1∶2560	1∶1280	≥1∶2560	1∶163840	
**419 S1**	2	NEG	D3	20	1∶320	1∶160	1∶40	1∶40	1∶640	S
**419 S4**	16	NEG		45	≥1∶2560	≥1∶2560	≥1∶2560	≥1∶2560	1∶40960	
**420 S1**	4	POS	D3	20	≥1∶2560	1∶640	1∶80	1∶160	1∶2560	S
**420 S4**	15	POS		22	≥1∶2560	≥1∶2560	≥1∶2560	≥1∶2560	1∶163840	
**428 S1**	3	NEG	D3	18	1∶1280	1∶1280	1∶40	1∶80	1∶2560	S
**428 S4**	15	NEG		54	≥1∶2560	≥1∶2560	≥1∶2560	≥1∶2560	1∶163840	
**434 S1**	5	NEG	D3	44	1∶1280	1∶640	1∶20	1∶80	1∶640	S
**434 S4**	13	POS		44	≥1∶2560	≥1∶2560	1∶1280	1∶640	1∶163840	
**435 S1**	5	NEG	D3	45	≥1∶2560	1∶640	1∶20	1∶40	1∶2560	S
**435 S3**	32	NEG		59	≥1∶2560	≥1∶2560	1∶1280	1∶1280	1∶163840	
**436 S1**	5	NEG	D3	48	≥1∶2560	≥1∶2560	1∶320	1∶40	1∶2560	S
**436 S3**	34	NEG		51	≥1∶2560	≥1∶2560	≥1∶2560	1∶320	1∶163840	
**463 S1**	4	NEG	D3	45	≥1∶2560	≥1∶2560	1∶160	1∶40	1∶10240	S
**463 S4**	16	NEG		55	≥1∶2560	≥1∶2560	≥1∶2560	≥1∶2560	1∶163840	
**465 S1**	5	NEG	D3	16	1∶40	1∶160	<1∶20	1∶20	1∶40	S
**465 S4**	14	POS		42	≥1∶2560	≥1∶2560	≥1∶2560	≥1∶2560	1∶163840	
**469 S1**	4	NEG	D3	43	1∶1280	1∶1280	1∶160	1∶320	1∶10240	S
**469 S4**	17	POS		45	≥1∶2560	≥1∶2560	≥1∶2560	1∶1280	1∶163840	
**481 S1**	5	NEG	D3	39	1∶640	1∶640	1∶40	1∶160	1∶10240	S
**481 S4**	16	POS		39	≥1∶2560	≥1∶2560	1∶1280	1∶1280	1∶163840	
**483 S1**	7	NEG	D3	4	<1∶20	<1∶20	<1∶20	<1∶20	1∶160	P
**483 S3**	18	POS		31	1∶40	1∶40	1∶160	1∶40	1∶2560	
**486 S1**	7	NEG	D3	2	<1∶20	<1∶20	<1∶20	<1∶20	<1∶40	P
**486 S3**	21	POS		29	1∶40	1∶20	1∶160	1∶40	1∶2560	
**496 S1**	5	NEG	D3	6	<1∶20	<1∶20	<1∶20	<1∶20	1∶160	P
**496 S4**	13	POS		24	1∶40	1∶40	1∶160	1∶40	1∶2560	
**497 S1**	5	NEG	D3	38	1∶640	1∶640	1∶80	1∶160	1∶10240	S
**497 S4**	12	NEG		40	≥1∶2560	≥1∶2560	1∶1280	≥1∶2560	1∶163240	
**498 S1**	8	POS	D3	7	<1∶20	<1∶20	1∶40	<1∶20	<1∶40	P
**498 S4**	15	POS		27	1∶80	1∶40	1∶320	1∶20	1∶2560	
**502 S1**	4	NEG	D3	33	1∶640	1∶640	1∶20	1∶80	1∶640	S
**502 S4**	12	POS		38	≥1∶2560	≥1∶2560	≥1∶2560	≥1∶2560	1∶163840	
**506 S1**	2	NEG	D3	32	1∶640	1∶640	1∶40	1∶160	1∶10240	S
**506 S4**	14	POS		45	≥1∶2560	≥1∶2560	1∶1280	≥1∶2560	1∶163840	
**521 S1**	6	POS	NEG	3	<1∶20	<1∶20	1∶40	<1∶20	<1∶40	P
**521 S4**	18	POS		34	1∶80	1∶8	1∶640	1∶40	1∶10240	
**523 S1**	3	NEG	D3	17	≥1∶2560	≥1∶2560	1∶320	1∶640	1∶640	S
**523 S2**	7	POS		32	≥1∶2560	≥1∶2560	≥1∶2560	≥1∶2560	1∶163840	
**524 S1**	4	NEG	D3	1	<1∶20	<1∶20	<1∶20	<1∶20	1∶160	P
**524 S4**	24	POS		32	1∶160	1∶160	1∶640	1∶160	1∶10240	
**527 S1**	2	NEG	D3	7	<1∶20	<1∶20	<1∶20	<1∶20	1∶160*	P
**527 S4**	10	POS		37	1∶160	1∶320	1∶640	1∶160	1∶655360	
**533 S1**	3	NEG	D3	1	<1∶20	<1∶20	<1∶20	1∶20	<1∶40	P
**533 S4**	10	POS		13	1∶40	1∶40	1∶320	1∶640	1∶2560	
**537 S1**	5	NEG	NEG	42	≥1∶2560	≥1∶2560	≥1∶2560	1∶1280	1∶10240	S
**537 S4**	12	POS		38	≥1∶2560	≥1∶2560	≥1∶2560	≥1∶2560	1∶163840	
**545 S1**	5	NEG	NEG	1	<1∶20	<1∶20	<1∶20	<1∶20	1∶160	P
**545 S4**	13	POS		25	1∶160	1∶320	1∶640	1∶640	1∶2660	
**546 S1**	11	POS	NEG	9	<1∶20	1∶20	1∶80	<1∶20	1∶640	P
**546 S3**	33	POS		33	1∶40	1∶80	1∶620	1∶40	1∶2560	
**547 S1**	11	NEG	NEG	1	<1∶20	<1∶20	<1∶20	<1∶20	1∶640	P
**547 S4**	33	POS		20	1∶80	1∶40	1∶640	1∶80	1∶2560	
**548 S1**	4	NEG	NEG	37	≥1∶2560	≥1∶2560	1∶320	1∶1280	1∶10240	S
**548 S2**	9	POS		42	≥1∶2560	≥1∶2560	≥1∶2560	≥1∶2560	1∶163840	
**553 S1**	8	POS	NEG	9	<1∶20	<1∶20	1∶80	<1∶20	<1∶40	P
**553 S4**	15	POS		26	1∶80	1∶40	1∶640	1∶40	1∶2560	
**554 S1**	4	NEG	NEG	2	<1∶20	<1∶20	<1∶20	<1∶20	1∶160	P
**554 S4**	18	POS		16	1∶160	1∶80	1∶320	1∶80	1∶10240	
**556 S1**	4	NEG	NEG	1	<1∶20	<1∶20	<1∶20	<1∶20	1∶160	P
**556 S4**	13	POS		25	1∶40	1∶40	1∶320	1∶40	1∶10240	
**559 S1**	3	NEG	D3	2	<1∶20	<1∶20	<1∶20	<1∶20	1∶160	P
**559 S4**	11	POS		26	1∶40	1∶80	1∶320	1∶80	1∶40960	
**564 S1**	3	NEG	NEG	4	<1∶20	<1∶20	<1∶20	<1∶20	1∶640	P
**564 S4**	11	POS		21	1∶40	1∶80	1∶320	1∶40	1∶10240	
**576 S1**	5	NEG	D3	7	<1∶20	<1∶20	<1∶20	<1∶20	1∶160	P
**576 S2**	7	POS		34	1∶40	1∶40	1∶160	1∶40	1∶10240	

N° days, number of days from the start of the symptoms; P, primary infection; S, secondary infection; NEG, negative; POS, positive; YFV, yellow fever virus; D3, DENV-3; RU, reference unit.

* discordant result.

#### Statistical analysis

Data analysis and plotting were carried out using the open-source R statistical package, Version 2.2.1 [20]. Multivariate regression analysis was performed by fitting a linear model using the R function lm, and p-values for trends, intercepts and interaction were obtained by the lm function from t-tests for the significance of the corresponding coefficients in the model. The F-test for the difference in variances was performed by the R function var.test. Linear discriminant analysis (LDA) was used to design a 2-D classifier, using as variables both the IgG unit level and the day of infection. LDA was coded in R directly from its definition in terms of group means and covariance matrices; e.g., see [21]. The bolstering resubstitution error estimation method used to assess the accuracy of the classifiers is based on the work described in [22]. Basically, it decreases the optimistic bias of the simple resubstitution error estimate (agreement-checking on the training data) by means of suitable bolstering probability density kernels placed at each training data point, producing a nearly-unbiased and low-variance estimator. The statistical analyses of test-set accuracy were performed in a blinded fashion, and the estimates were based on counting the number of correctly classified test samples and dividing by the total number of test samples. Confidence intervals for the test-set estimates were obtained from the binomial distribution, using the R function binom.test. The R code for LDA and bolstered resubstitution error estimation is provided as supplementary material (Statistical package S1) and is available for downloaded at this journal site.

## Results

**Figure 1:**
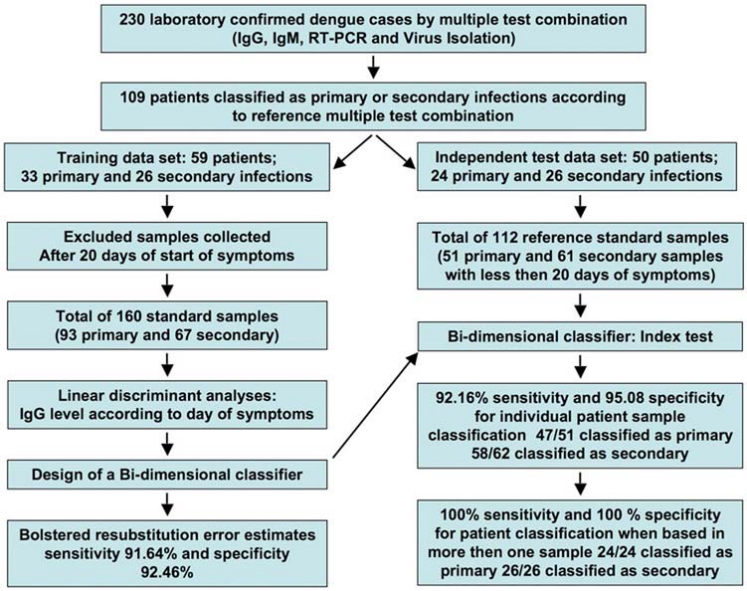
Data flow for standard samples from the cohort of dengue fever patients. Two to five blood samples were obtained from the patients on different days; these samples were pooled, resulting in 119 primary infection samples and 81 secondary infection samples, for a total of 200 samples. The effective training data set consisted of all available training samples from day 20 or earlier (93 primary and 67 secondary samples, for a total of 160). After design of the 2-D classifier, its accuracy was assessed both by training set bolstered error estimates and independent test-set error estimates. For the test set, the available samples were pooled, and those obtained after 20 days from the self-reported onset of symptoms were eliminated from consideration. The resulting test set had 51 primary and 61 secondary samples, for a total of 112 samples. The test set was also used to assess the accuracy of a diagnostic classifier that used all available samples for each patient.

Samples from 109 well-defined dengue infection cases, comprising 54 male and 55 female volunteers, were used in this work as reference samples. Summary of the demographic characteristics of these patients is presented in the Table 1. Samples from 59 patients were selected as the training set; of these, 33 presented primary infection, and 26 presented secondary infection, according to the “Recife” classification method, as defined in the methods section. A separate independent test set was established using data from the remaining additional 50 patients; of these, 24 corresponded to primary and 26 to secondary infection, again according to the Recife method. A schematic flow chart outlining the data processing steps is depicted in Figure 1.

The 59 patients of the training set provided two to five blood samples collected on different days. After about day 20, it is not possible to distinguish the IgG responses from the primary and secondary infection samples. Therefore, we limited our analysis to samples taken < = 20 days from the start of symptoms. For the training set, this resulted in 93 primary infection samples and 67 secondary infection samples, for a total of 160 training samples (Figure 1).

**Figure 2:**
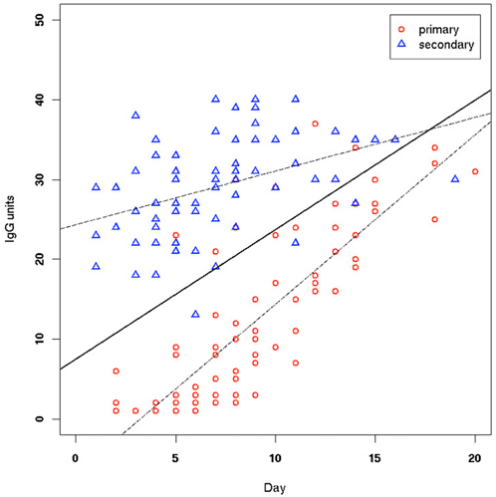
Training data, with primary and secondary infection classification according to the CPqAM criteria, with regression lines (dashed lines) and LDA classifier (solid line) superimposed. The regression line for the primary infection group corresponds to the equation IgG = −6.865+2.126×day, whereas the one for the secondary infection corresponds to IgG = 24.327+0.674×day. The slopes (dependence on day of infection) and intercepts were highly significant, for both primary and secondary infection groups (p<0.0005 in all cases). The difference between the intercepts is also highly significant (p<10-15), indicating a difference in initial immune response that reflects immunological memory in the case of the dengue-specific IgG. In addition, there is significant interaction between infection type and days of fever (p<10-7), that is, the lines are not parallel, and the trends within each group are significantly different, suggesting that it may be possible to define acute dengue infections on the basis of the rise in IgG level alone. The two regression lines converge near the 20-day limit, indicating that the groups cannot be reliably discriminated beyond this number of days. The equation for the LDA line is y = 7.494+1.623×x. The LDA classifier is depicted as a solid line in Figure 2.

Figure 2 shows a plot of IgG unit values versus number of days of symptoms for the 160 samples in the training data set. A multivariate linear model was fitted to this data, with IgG units as the dependent variable being regressed on days of fever and infection type (primary/secondary). The fitted line for the primary infection group (red circles) gave IgG = −6.865+2.126×day, whereas the one for the secondary infection group (blue triangles) gave IgG = 24.327+0.674×day. The regression lines are depicted as dashed lines in Figure 2, superimposed on the training data. The primary infection samples began with low IgG levels that quickly rose with time, whereas the secondary infection samples began with a nonzero basal value (reflecting immunological memory) and raised little over time. The slopes (dependence on day of infection) and intercepts were highly significant, for both primary and secondary infection groups (p<0.0005 in all cases). There were very few outliers. An F-test to compare the variances in the two groups does not reject the hypothesis that they are identical therefore the multivariate model allows us to test jointly the two lines. We find that the difference between the intercepts is highly significant (p<10^−15^), indicating a difference in initial immune response that reflects immunological memory in the case of the dengue-specific IgG. In addition, there is significant interaction between infection type and days of symptoms (p<10^−7^), that is, the lines are not parallel, and the trends within each group are significantly different, suggesting that it may be possible to define acute dengue infections on the basis of the rise in IgG level alone; however, this is not the goal of this study. The two regression lines converge near the 20-day limit, which is another indication that the groups cannot be reliably discriminated beyond this number of days. Linear Discriminant Analysis (LDA) was performed to obtain a linear classifier based on these data (the outlier samples were not excluded). The equation for the LDA line is y = 7.494+1.623×. The LDA classifier is depicted as a solid line in Figure 2.

**Figure 3:**
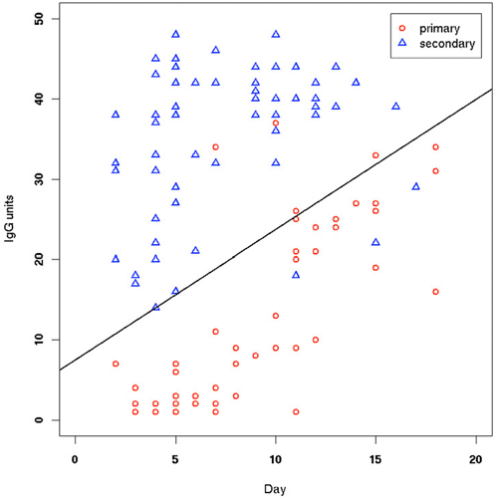
Independent test data, with primary and secondary infection classification according to the CPqAM criteria, with a solid line representing the previously designed classifier, for inspection. Test data were obtained from 50 additional patients in the cohort. As with the training data, all quantified samples were pooled and those that had been obtained more than 20 days after the onset of symptoms were ignored, resulting in 51 primary and 61 secondary infection samples, according to the CPqAM criteria, for a total of 112 test samples.

Table 3 displays some of the threshold values calculated using the designed classifier equation (y = 7.494+1.623×) for the IgG units used to classify samples as indicating primary infection (below the threshold) or secondary infection (above the threshold), as a function of sample collection day. As expected, the threshold values increase over time. Sensitivity and specificity for this classifier were estimated via two methods. First, we used the training data itself to derive bolstered resubstitution error estimates (see Methods section). The estimated sensitivity and specificity found were 91.64% and 92.46%, respectively. The classifier recommended by the manufacturer corresponds to a horizontal line at IgG unit threshold = 40. This did not perform satisfactorily at all on our data, as this classifier is completely non-specific (this can be seen in Figure 2, which shows that almost all of the secondary infection IgG responses are below 40, not above). Decreasing the IgG unit level from the recommended value of 40 to the optimal one-dimensional classifier improves accuracy, but not to an acceptable level (data not shown). This underscores the need to include the stage (day) of disease as a classification variable, as in the proposed LDA classifier, to account for rising levels of IgG response. Secondly, the designed LDA classifier was tested on an independent set of samples, obtained from 50 additional patients, as described earlier (Figure 1). This typically results in a more accurate error estimator than the one using the training samples, provided the number of test samples is large. As before, we pooled all quantified sample data and ignored those that had been obtained more than 20 days after the onset of symptoms, resulting in 51 primary and 61 secondary infection samples, for a total of 112 test samples. This test set is large enough to allow accurate estimates of classification accuracy. Figure 3 displays the data for these samples, overlapped on the proposed classifier for inspection.

**Table 3:** Threshold values for the IgG units reference values used to classify samples into primary infection (below the threshold) or secondary infection (above the threshold), as a function of sample day, according to the designed LDA classifier.

Days of symptoms onset	IgG Unit Threshold
0	7.5
2	10.7
4	14.0
6	17.2
8	20.5
10	23.7
12	27.0
14	30.2
16	33.5
18	36.7
20	40.0

Based on this independent test set of 112 samples, the estimated sensitivity and specificity of the proposed classifier, using as groundtruth the results of the Recife method, were 92.16% (95% CI = 81.12 to 97.82) and 95.08% (95% CI = 86.29 to 98.97), respectively. These accuracy estimates are not appreciably different from those obtained previously by bolstered resubstitution. The proposed classifier was able to correctly predict 47 of the 51 primary samples and 58 of the 61 secondary samples. We also used the independent test data to break down the estimates of accuracy according to various ranges of days of infection (Table 4). The results were consistent with the expectation that classification between primary and secondary infection would be easier early in the infection than at later times. The proposed classifier performed perfectly with samples taken on day 4 or earlier, but its sensitivity and sensibility decreased with time.

**Table 4:** Accuracy of the proposed LDA classifier according to ranges of days of symptoms, based on the independent test set, with 95% confidence interval limits.

Days of Symptoms	Sensitivity	Specificity
1–4	100.00 (66.37–100.00)	100.00 (80.49–100.00)
5–8	94.44 (72.71–99.86)	100.00 (78.20–100.00)
9–12	84.62 (54.55–98.08)	95.24 (76.18–99.88)
13–20	90.91 (58.72–99.77)	75.00 (34.91–96.81)
≤20	92.16 (81.12–97.82)	95.08 (86.29–98.97)

As a final test, the serum samples from the independent test set were also classified according to the hemagglutination inhibition (“HI”) method and to an in-house IgG-captrue ELISA (“Rio”) method [13], as defined in the Methods section, aiming to characterize the serological immune response and classify the infections as primary or secondary. From the panel of 50-paired sera, 24 cases were characterized as primary cases and 26 as secondary ones by the HI method (Table 2). Complete (100%) agreement was observed between the proposed classifier and the HI method (WHO criteria) in the classification of primary and secondary dengue infections. The Rio method identified 26 secondary infections, 22 primary infections and 2 inconclusive cases. Using these results as the groundtruth, we obtained an overall accuracy of 96% (48/50) for the proposed classifier. Using the HI method as groundtruth, the Rio method was more sensitive in confirming secondary cases (100%, 26/26) than primary ones (92%, 22/24). There was one instance where both the Recife and HI method called one patient as primary infection, whereas the call according to the Rio method was secondary infection.

## Discussion

In this manuscript, we describe a process for designing and validating a classification method to discriminate between primary and secondary dengue infections based on IgG antibody levels and the number of days of symptoms, and we demonstrate that a 2-D classifier designed using this approach is very reliable. We make available software that enables the user to insert their IgG data for the training and test data sets from their standard samples and obtain a validated 2-D classifier, which in our study generates classifications identical to the ones made by the HI assay.

Although secondary infection with dengue virus is the most widely accepted risk factor for the development of dengue hemorrhagic fever, there is no simple, rapid, and reliable method that can routinely be used to discriminate between primary and secondary infections in the early days of an infection. Distinguishing between primary and secondary infections can be of great importance, particularly in endemic areas in which the dengue virus has recently arrived and primary infections are also frequent. Moreover, laboratory confirmation of acute dengue infection can sometimes be difficult, depending on the how many days the person has been sick and what diagnostic tests are available. It is important to note that the proposed 2-D classifier is not intended to determine the presence of an acute dengue infection, but to classify an acute infection as primary or secondary. In our experience, the ideal combination of tests to detect an acute infection in the first 5 days of symptoms is the use of RT-PCR associated with IgG serology to classify infection history, and from the sixth day of symptoms and after, the use of IgM and IgG serology.

The most commonly used serological test is IgM-capture ELISA. Nevertheless, this test is not sufficiently sensitive during the first 3–5 days of symptoms. In primary cases, both IgM and IgG antibody detection often will give negative results during this period. Thus, in these early days of disease a diagnosis will only be possible by RT-PCR, virus isolation and/or dengue NS1 antigen detection by ELISA. It should be pointed out that in some secondary dengue infections, specific IgM is often not detected at all.

The hemagglutination inhibition assay has been the gold standard for the serological diagnosis of dengue infection, as well as to classify the patient's dengue immune response [7]. However, the most reliable way to define primary and secondary dengue infection is based on a combination of multiple laboratory tests (virus isolation and/or detection of virus RNA by PCR, IgM and IgG antibody detection) performed on blood specimens collected at two time points, at least. A primary dengue infection is defined as the absence of specific anti-dengue IgG antibodies in the first serum samples during the acute phase, with anti-dengue IgM, virus isolation and/or virus RNA being present, and dengue virus IgG being detected in a later sample. In contrast, secondary dengue infection is defined by the presence of specific anti-dengue IgG and the absence of anti-dengue IgM in the first sample, together with a positive RT-PCR and/or virus isolation, followed by the presence of anti-dengue IgM in a later sample. Defining primary and secondary dengue infections by means of these rigorous criteria is very expensive, and most clinical laboratories in dengue-endemic countries cannot realistically perform all these assays on all of their samples. Moreover, even when all the assays are available, because of the dates of the blood collections and the immunological windows, it is not always possible to unambiguously define primary versus secondary dengue infections. The main caveat is that depending on how long the patient is sick before the first sample is collected, it may be possible to detect anti-dengue specific IgG at the time of the first medical visit in a primary dengue infection. Furthermore, classification of primary versus secondary dengue immune responses exclusively on the basis of the HI test, following the WHO criteria [7] it is not always reliable and can be misleading [6]. In this case, diagnosis is based on the antibody titers of paired serum samples, and cross-reactivity among flaviviruses is common and can lead to false results. In addition, during acute secondary dengue infection, pre-existing serotype-specific antibodies are boosted [4], and if the number of days since the onset of symptoms is not taken into account, the HI test can result in misleading classification. In contrast, in a separate study we have found no evidence of cross-reactivity of the PanBio dengue IgG ELISA with sera from with Yellow Fever 17DD vaccinated volunteers (Table S1). Six of the 32 Yellow fever vaccinees presented dengue-specific IgG 45 to 90 days after the vaccination, however it was later confirmed that those individuals had natural dengue infection. Cross-reactivity among flavivirus diagnostic kits are common and it is important to select a dengue-IgG kit with minimum cross-reactivity with other local flavivirus, however differentiating cross-reactivity against natural infection may not be an easy task in endemic areas. If possible, it is important actually verify with standard samples collected locally.

Thus, a simple alternative laboratory method for the classification of primary and secondary dengue antibody responses is highly desirable. Matheus et al. [12] developed an IgG avidity test to discriminate between primary and secondary dengue virus infection using a single acute-phase serum sample and claimed good sensitivity and specificity. However, the real performance of this method still need to be evaluated in independent reference standard samples from patients for whom the classification of serological response was based on criteria other than only the HI test. The results from our dengue cohort clearly support the contention that to correctly delineate primary and secondary responses, it is strictly necessary to combine several assays, such as IgM and IgG levels, virus isolation and/or viral RNA detection. For example, an absence of IgM in some secondary cases, even in later samples, was seen in several of our dengue cohort patients, and this phenomenon has also been observed by others [23]. Dengue infections could be characterized as primary or secondary by determining the ratio of units of dengue IgM to IgG antibody [23]. However there are many cases where IgM is undetectable or not yet present and this criterion could not be applied. It would be necessary another test to confirm the acute dengue infection, for example, a positive RT-PCR, which is the most sensitive method to confirm dengue infection at the early days of the disease.

Because IgM and IgG dengue ELISAs kits are commercially available at relatively low cost, dengue fever diagnosis is now being done in many laboratories worldwide. In this study we took advantage of the existence of a good commercial IgG-ELISA kit to develop a 2-D classifier, using IgG levels and self-reported days of symptoms from a cohort of 109 patients with well-characterized primary or secondary dengue infections. This approach would allow to define, according to how many days of symptoms, what levels of anti-dengue IgG would be compatible with primary or secondary infections.

We have found, by using multiple accuracy estimation methods, that the sensitivity and specificity of the designed 2-D LDA classifier are vastly superior to the most commonly used stage-independent 1-D classifiers (data not shown). For individual sample classification, estimates of sensitivity and specificity of the 2-D classifier were in the range of 90–95%. For patient classification using a majority-voting rule, independent test-set estimates of both sensitivity and specificity were 100%.

The 2-D LDA classifier was tested in an independent set of 50 patients and the results compared with two other methods, the HI assay, according to WHO guidelines [7] and an in-house made ELISA. There was a total 100% agreement between the HI results and our 2-D LDA classifier. When those results were compared to the in-house IgG-ELISA (“Rio” method) described by Miagostovich et al. [13], the overall sensitivity was 96% (48/50). As expected, the Rio method was more sensitive in confirming secondary cases (100%, 26/26) than primary ones (92%, 22/24). In primary infections, the Rio method is generally negative in the first week after the onset of the disease and individual variation may occur. Therefore, for a definitive and reliable result using the Rio method, it is important in some cases to also use a second sample from the convalescence phase. However when used samples from the acute and convalescent phase the results are clear, because the Rio titers in acute samples are low (up to day 5 after the onset of the disease) and very high in convalescent sera from secondary dengue cases as previously described by Miagostovich et al. [13]. These two independent tests results corroborated our immune response classification results (Table 2).

The classifier developed in this study is currently being used, in daily practice, in our laboratory and has shown excellent performance on independently validated data that is compatible with the results presented here. Indeed, in our on going dengue study the most reliable and cost efficient combination of diagnostic exam is the detection of anti-dengue IgM, IgG and RT-PCR. With this combination of tests we can determine in 100% of the cases the presence of acute dengue infection, the viral serotype and with the use of the 2-D classifier, the patient serological history within 24 hours of the first blood sample collected.

It is noteworthy that very few primary and secondary standard samples were dispersed among samples of the other type (Figures 2 and 3), suggesting also that patient-reported number of days of dengue symptoms, although a subjective measure, is considerably more accurate than often acknowledged. We would also like to point out that the methodology described here can be employed to design classifiers based on results from kits other than the PanBio kit or in-house assays such as the one in the Rio method, as long as a good set of standard primary and secondary reference samples is available.

According to the manufacturer of the PanBio kit, and as found by Vaughn [15], an IgG result of 40 PanBio units correlates with an HI titer of ≥1∶1280, the cut-off used to distinguish between primary and secondary dengue infection based on WHO criteria [7]. Thus, using this criteria, a result of >40 IgG units can be used to identify a secondary infection and IgG units of >11–40 to detect a primary infection. However, we have shown that these suggested values do not provide reliable classification results in patients from our cohort, nor does any stage independent 1-D classifier that does not take in consideration when a sample was collected in relation to the onset of symptoms.

In conclusion, laboratories in endemic areas interested in distinguishing acute primary dengue infections from acute secondary infections can use the methodology we have described here. These laboratories can collect and characterize a set of reference samples from acutely ill patients in their region, and if necessary some of the characterization assays may be carried by another laboratory; they can then design a reliable 2-dimensional classifier based only on the IgG levels quantified by a clinical assay kit and the number of days of symptoms reported by the patient. In the supplemental material we provide for download R software for the calculation of the LDA classifier, as well as the bolstered resubstitution error estimator, and instructions that other laboratories can use to design their classifiers (Statistical Package S2). We ask the users of this classifier to share the classification data, classifier performance and standard samples with other investigators using the PLoS One post-publication and communication tools. By applying this classifier in samples from other cohorts and sharing the results we can further strengthen the validation and define the value of this method. In addition, we will offer and make available in our laboratory web site an interface to a set of dengue diagnostic tools and database (http://augustlab.bs.jhmi.edu/index.html). The use of this approach can allow clinicians to more quickly and reliably identify whether their patients are experiencing a primary or secondary dengue infection, which allows the assessment of risk of developing DHF in order to decide what is the most appropriate care and also reduce cost by reducing hospitalizations worldwide.

## Supporting Information

Table S1 Yellow fever cross-reaction supplemental data
(0.08 MB DOC)

Statistical package S1 MathLab “R” code for calculation of 2-LDA and error estimation
(0.01 MB ZIP)
