# Supplementary material for: Reliable Classifier to Differentiate Primary and Secondary Acute Dengue Infection Based on IgG ELISA
Source: PLoS One. 2009 Apr 2;4(4):e4945. doi: 10.1371/journal.pone.0004945 (PMC2660412; doi:10.1371/journal.pone.0004945)
Supplement: Table S1 — Yellow fever cross-reaction supplemental data (0.08 MB DOC) [file pone.0004945.s001.doc]

Cohort Yellow Fever

| **Subject Nº** | **Dengue IgG (UR PanBio)** | | **Yellow Fever IgG (GAC-ELISA)** | | YELLOW Fever - PRNT | |
| --- | --- | --- | --- | --- | --- | --- |
|  | **Pre- YF vaccine** | **Post-YF vaccine** | **Pre- YF vaccine** | **Post-YF vaccine**  **(Number of days)** | **Pre- YF vaccine** | **Post-YF vaccine** |
| **5** | Negative | Negative | Negative | Positive ( 70 ) | <1:20 | 1:1280 |
| **17** | Negative | Negative | Negative | Positive ( 90 ) | <1:20 | 1:1280 |
| **18** | Negative | Negative | Negative | Positive ( 85 ) | <1:20 | 1:1280 |
| **44** | Negative | Negative | Negative | Positive ( 65 ) | <1:20 | 1:640 |
| **48** | Negative | Negative | Negative | Positive ( 90 ) | <1:20 | 1:1280 |
| **53** | Negative | Negative | Negative | Positive ( 90 ) | <1:20 | 1:640 |
| **60** | Negative | Negative | Negative | Positive ( 70 ) | <1:20 | 1:640 |
| **71** | Negative | Negative | Negative | Positive ( 90 ) | <1:20 | 1:1280 |
| **90** | Negative | Negative | Negative | Positive ( 80 ) | <1:20 | 1:1280 |
| **107** | Negative | Negative | Negative | Positive ( 70 ) | <1:20 | 1:1280 |
| **122** | Negative | Negative | Negative | Positive ( 40 ) | <1:20 | 1:1280 |
| **134** | Negative | Negative | Negative | Positive ( 40 ) | <1:20 | 1:1280 |
| **145** | Negative | Negative | Negative | Positive ( 30 ) | <1:20 | 1:640 |
| **161** | Negative | Positive | Negative | Positive ( 30 ) | <1:20 | 1:640 |
| **166** | Negative | Positive | Negative | Positive ( 90 ) | <1:20 | 1:1280 |
| **174** | Negative | Positive | Negative | Positive ( 90 ) | <1:20 | 1:1280 |
| **179** | Negative | Positive | Negative | Positive ( 90 ) | <1:20 | 1:1280 |
| **194** | Negative | Negative | Negative | Positive ( 70 ) | <1:20 | 1:1280 |
| **198** | Negative | Negative | Negative | Positive ( 70 ) | <1:20 | 1:640 |
| **206** | Negative | Positive | Negative | Positive ( 45 ) | <1:20 | 1:640 |
| **208** | Negative | Positive | Negative | Positive ( 45 ) | <1:20 | 1:640 |
| **209** | Negative | Negative | Negative | Positive ( 60 ) | <1:20 | 1:1280 |
| **210** | Negative | Negative | Negative | Positive ( 40 ) | <1:20 | 1:640 |
| **213** | Negative | Negative | Negative | Positive ( 60 ) | <1:20 | 1:1280 |
| **217** | Negative | Negative | Negative | Positive ( 40 ) | <1:20 | 1:1280 |
| **218** | Negative | Negative | Negative | Positive ( 30 ) | <1:20 | 1:640 |
| **220** | Negative | Negative | Negative | Positive ( 40 ) | <1:20 | 1:1280 |
| **223** | Negative | Negative | Negative | Positive ( 30 ) | <1:20 | 1:640 |
| **230** | Negative | Negative | Negative | Positive ( 50 ) | <1:20 | 1:1280 |
| **232** | Negative | Negative | Negative | Positive ( 30 ) | <1:20 | 1:640 |
| **243** | Negative | Negative | Negative | Positive (45 ) | <1:20 | 1:1280 |
| **247** | Negative | Negative | Negative | Positive | <1:20 |  |
